# Downregulation of senescence-associated secretory phenotype by knockdown of secreted frizzled-related protein 4 contributes to the prevention of skin aging

**DOI:** 10.18632/aging.204273

**Published:** 2022-09-07

**Authors:** Kento Takaya, Toru Asou, Kazuo Kishi

**Affiliations:** 1 Department of Plastic and Reconstructive Surgery, Keio University School of Medicine, Shinjuku, Tokyo, Japan

**Keywords:** skin, fibroblast, SASP, SFRP4

## Abstract

There is growing evidence that the appearance and texture of the skin that is altered during the aging process are considerably enhanced by the accumulation of senescent dermal fibroblasts. These senescent cells magnify aging via an inflammatory, histolytic, and senescence-associated secretory phenotype (SASP). Secreted frizzled-related protein 4 (SFRP4) was previously determined to be expressed in dermal fibroblasts of aging skin, and its increased expression has been shown to promote cellular senescence. However, its role in the SASP remains unknown. We found that SFRP4 was significantly expressed in p16ink4a-positive human skin fibroblasts and that treatment with recombinant SFRP4 promoted SASP and senescence, whereas siRNA knockdown of SFRP4 suppressed SASP. Furthermore, we found that knockdown of SFRP4 in mouse skin ameliorates age-related reduction of subcutaneous adipose tissue, panniculus carnosus muscle layer, and thinning and dispersion of collagen fibers. These findings suggest a potential candidate for the development of new skin rejuvenation therapies that suppress SASP.

## INTRODUCTION

The skin is the outermost protective barrier of an organism and is composed of two main layers: the epidermis and dermis. The dermis lies beneath the epidermis and plays an important role in the structure and function of the skin [1]. It primarily consists of an extracellular matrix (ECM) generated by numerous fibroblasts [2]. Dermal fibroblasts are actively involved in the immune response of the skin, wound healing, and communication with the nervous and vascular systems [3–5]. Interestingly, it has been suggested that aging fibroblasts may lead to loss of function or dysfunction [6, 7] and may even contribute to visible clinical signs of aging skin, such as decreased swelling pressure and increased wrinkles [8]. Emerging hypotheses of skin aging postulate that senescence of fibroblasts primarily promotes skin fading and aging by irreversibly arresting proliferation and enhancing the release of the senescence-associated secretory phenotype (SASP) [9, 10].

The SASP induces chronic inflammation via chemokines and proinflammatory factors, inhibits proliferation by impairing the release of essential growth factors, and promotes ECM degradation by enhancing the activation of proteolytic enzymes, including matrix-degrading metalloproteinases [11]. Human fibroblast-derived SASP consists of increased concentrations of interleukins, matrix metalloproteinases (MMPs), and various chemokines, among many other factors [12, 13]. These secretions contribute to the progression of various age-related diseases [14], malignancies including squamous cell carcinoma [15, 16], prolonged wound healing, and skin aging [17]. In particular, human fibroblasts isolated from aged skin express secreted proteins related to inflammation and apoptosis, such as tumor necrosis factor alpha (TNF-α) [18], in addition to the usual SASP, called skin aging-associated secreted proteins (SAASP) [19].

Furthermore, it has been previously shown that SASP-induced fibroblasts can spread to neighboring non-aging fibroblasts [20]. This has attracted attention to the development of anti-aging therapies that suppress the SASP and selectively eliminate senescent cells. It has been reported that p16^ink4a^ functions as a specific marker of senescent cells [9], and *in vivo* depletion of p16^ink4a^-positive senescent cells significantly improves organ function in aging organisms by suppressing the SASP, extending both healthy and overall life span [21, 22]. However, it is unclear whether SASP suppression of senescent cells in the dermis delays aging.

Previous studies have shown through RNA sequencing that secreted frizzled-related protein 4 (SFRP4) mRNA and protein expression levels are significantly upregulated with age in human fibroblasts and that external supplementation of SFRP4 promotes fibroblast senescence [23]. SFRP4 is an extracellular Wnt antagonist that fine-tunes its signaling activity by binding directly to Wnt [24]. Although there are few descriptions of the association between SFRP4 and aging, it has been noted that SFRP4 expression is increased in scleroderma, correlates with skin and lung fibrosis, and may serve as a biomarker for epithelial mesenchymal transition [25].

We investigated the classical model of skin fibroblasts based on Hayflick’s mitotic limit [26], the observation of SFRP4 expression in replicating senescent cells, and the effect of regulating this on the suppression of SASP and aging skin. These results may contribute to the development of new therapies to ameliorate skin aging.

## RESULTS

### Upregulated SFRP4 expression in aging skin fibroblasts

**Figure 1:**
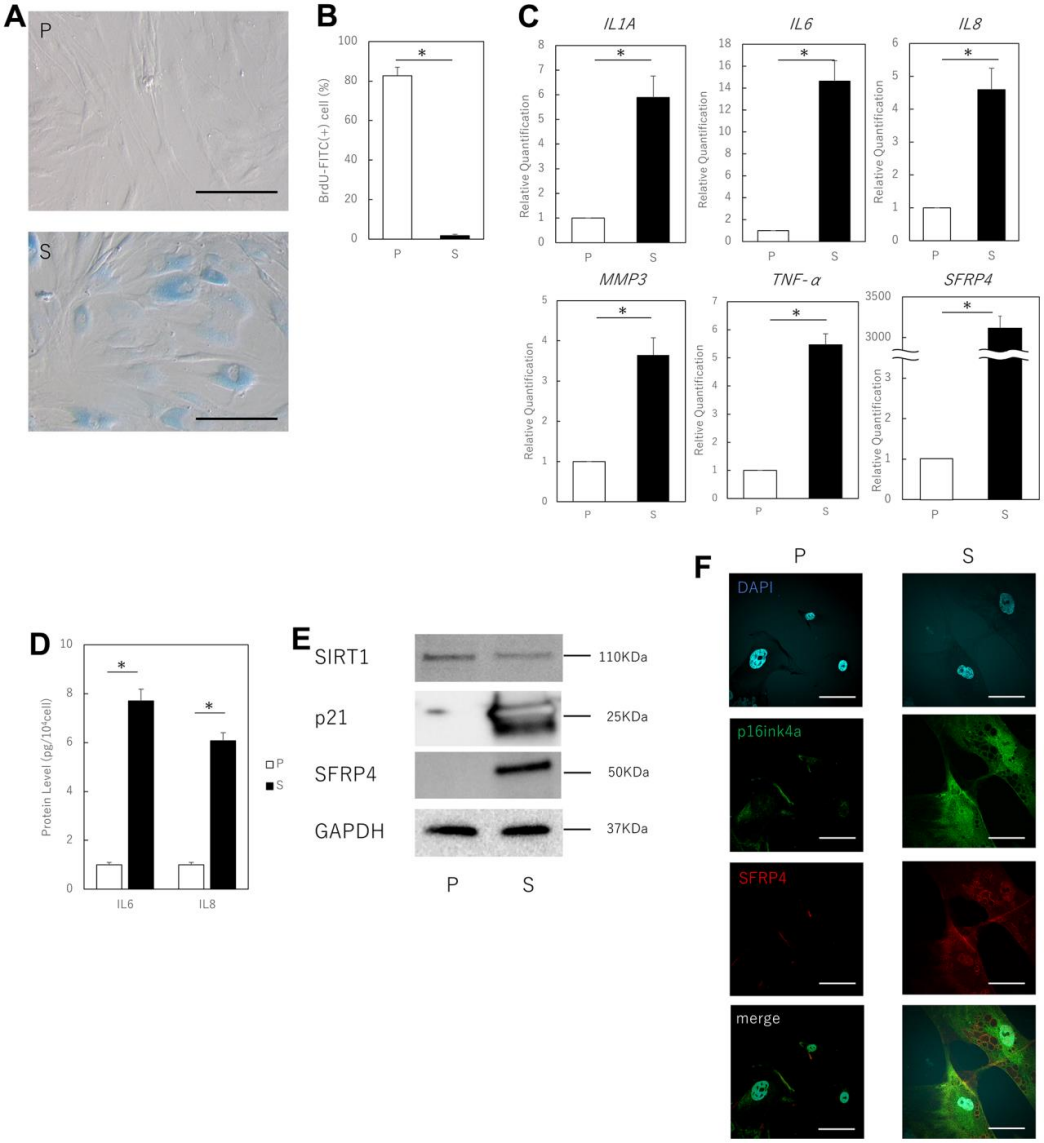
**Aging human skin fibroblasts express SFRP4.** (**A**) SA-β-gal staining of proliferating and senescent cells. Senescent cells profusely express SA-β-gal in the cytoplasm. Bar = 50 μm. (**B**) BrdU absorption by proliferating and senescent cells. Senescent cells have decreased BrdU absorption due to decreased mitotic activity. (**C**) RT-PCR analysis of SASP gene expression by cell extracts. GAPDH was used as the housekeeping gene. (**D**) ELISA for protein expression of IL6 and IL8. (**E**) Western blot analysis of cell extract proteins. GAPDH was used as the housekeeping protein. (**F**) Immunostaining of p16^ink4a^ and SFRP4 in proliferating and senescent cells. SFRP4 expression is observed in the cytoplasm in senescent cells consistent with p16ink4a positivity. Bar = 20 μm. P: Proliferating cells. S: Senescent cells. *; P < 0.05. Mann–Whitney U test was used to analyze differences between two groups. All experiments were repeated in triplicate.

We used previously reported methods to confirm that aged human skin fibroblasts that had undergone several replications met the characteristics of senescent cells. These cells were simultaneously examined to assess whether SFRP4 expression was upregulated. Replication-aged fibroblasts showed a characteristic flattened and expanded morphology and increased SA-β-gal activity, as previously shown [9] (Figure 1A). Furthermore, analysis of BrdU uptake showed that proliferative activity was significantly lower in senescent cells than in proliferating cells (young cells) (P = 0.000005) (Figure 1B). Furthermore, real-time polymerase chain reaction (PCR) showed that the expression levels of characteristic fibroblast SASP factors, such as IL1A (P = 0.03), IL6 (P = 0.018), IL8 (P = 0.031), MMP3 (P = 0.025), and TNF-α (P = 0.022) were elevated in replication-aged fibroblasts compared to proliferating cells, and SFRP4 was also markedly elevated (P = 0.0018) (Figure 1C). ELISA showed that IL6 (P = 0.028) and IL8 (P = 0.021) protein expression was significantly enhanced in senescent cells (Figure 1D). Western blotting results showed that the protein expression level of SFRP4 was higher in senescent cells than in proliferating cells. Senescent cells also had increased expression of p21, an established senescence marker that functions as a cell cycle inhibitor by blocking G1/S-mediated progression when associated with cyclin-dependent kinase 2. In addition, senescent cells showed decreased expression of SIRT1, which protects cells from replicative senescence by promoting telomerase reverse transcriptase transcription (Figure 1E). Cell immunostaining showed that SFRP4, which is rarely expressed in proliferating cells, was significantly expressed in p16^ink4a^-positive senescent cells (Figure 1F).

### Control of SFRP4 expression regulates SASP

**Figure 2:**
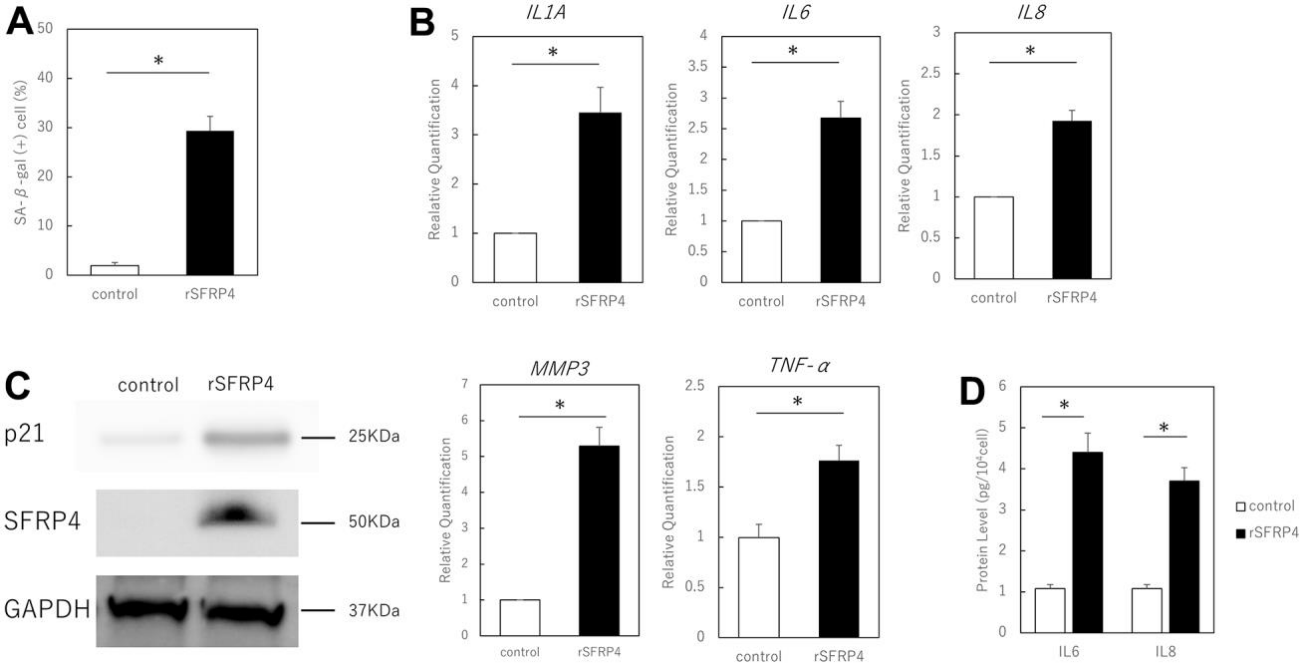
**Effect of recombinant SFRP4 (rSFRP4) on SASP.** (**A**) Comparison of the percentage of SA-β-gal-positive cells. rSFRP4 treatment increased SA-β-gal expression in proliferating cells. (**B**) RT-PCR analysis of the SASP gene expression. GAPDH was used as the housekeeping gene. (**C**) ELISA for protein expression of IL6 and IL8. (**D**) Western blot analysis of the effects of SFRP4 administration on confirmation and senescence. rSFRP4 treatment was observed to increase SASP at the mRNA and protein levels. rSFRP4; recombinant SFRP4. *; P < 0.05. Mann–Whitney U test was used to analyze differences between two groups. All experiments were performed in triplicate.

To investigate the effect of SFRP4 on the SASP, we added recombinant SFRP4 to the culture medium. We verified cellular protein uptake from the medium by measuring SFRP4 levels in fibroblasts maintained in a medium containing recombinant SFRP4. Cellular protein uptake was determined by western blot protein bands or real-time PCR. Treatment of proliferating human skin fibroblasts for 10 days with 15 μg/ml of human rSFRP4 revealed an increase in SA-βGal-positive cells in some cells, but not all (P = 0.012) (Figure 2A). RT-PCR analysis also showed that the expression of the SASP factors IL1A (P = 0.041), IL6 (P = 0.025), IL8 (P = 0.021), MMP3 (P = 0.037), and TNF-α (P = 0.033) was significantly increased by rSFRP4 treatment (Figure 2B). ELISA showed that IL6 (P = 0.029) and IL8 (P = 0.021) protein expression was significantly enhanced by rSFRP treatment (Figure 2C). p21 protein expression levels were also increased by rSFRP4 treatment, and SFRP4 uptake was also observed (Figure 2D).

**Figure 3:**
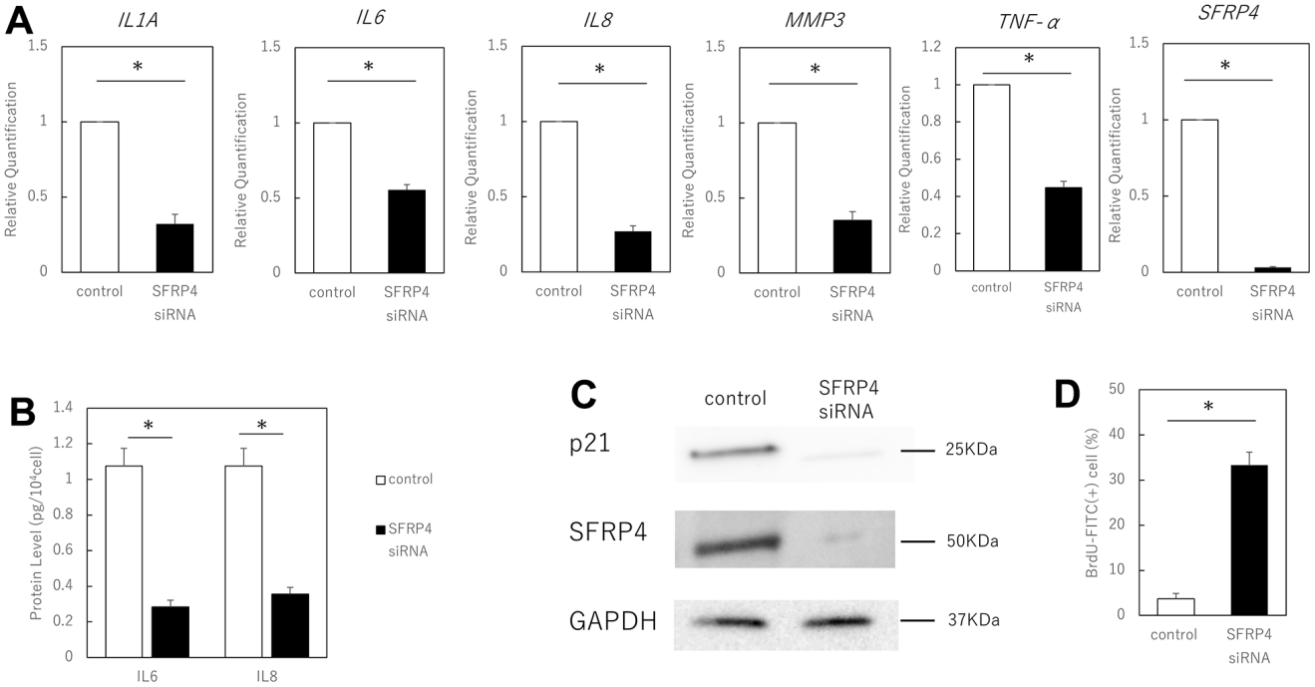
**Effect of SFRP4 siRNA knockdown on senescent cells.** (**A**) RT-PCR analysis of SASP gene expression. GAPDH was used as the housekeeping gene. (**B**) ELISA for protein expression of IL6 and IL8. (**C**) Western blotting analysis to confirm SFRP4 knockdown and its effect on senescence. SFRP4 knockdown induced decreased expression of SASP factors and senescence-related proteins. (**D**) Increased BrdU absorption after SFRP4 siRNA transfection. Knockdown of SFRP4 resulted in the restoration of proliferative capacity. *; P < 0.05. Mann–Whitney U test was used to analyze differences between two groups. All experiments were performed in triplicate.

We further tested whether SFRP4 contributes to the induction of senescence by silencing SFRP4 in aging skin fibroblasts and by evaluating senescence markers. Following strong silencing of SFRP4 at 72 h after transfection with SFRP4 siRNA, RT-qPCR analysis revealed that gene expression levels of the SASP factors IL1A (P = 0.0086), IL6 (P = 0.0028), IL8 (P = 0.006), MMP3 (P = 0.0084), TNF-α (P = 0.0078) and SFRP4 (P = 0.0000034) were significantly reduced in the SFRP4 siRNA group (Figure 3A). ELISA showed that IL6 (P = 0.011) and IL8 (P = 0.017) protein expression was significantly suppressed by SFRP knockdown treatment (Figure 3B). Western blot analysis revealed a marked reduction in the levels of p21 (Figure 3C). In addition, SFRP4 silencing promoted significant BrdU uptake (P = 0.0025) (Figure 3D), further indicating that SFRP4 contributes to the growth inhibition characteristic of senescence. In summary, our results indicate that SFRP4 promotes fibroblast senescence.

### Knockdown of SFRP4 inhibits skin aging in mice

**Figure 4:**
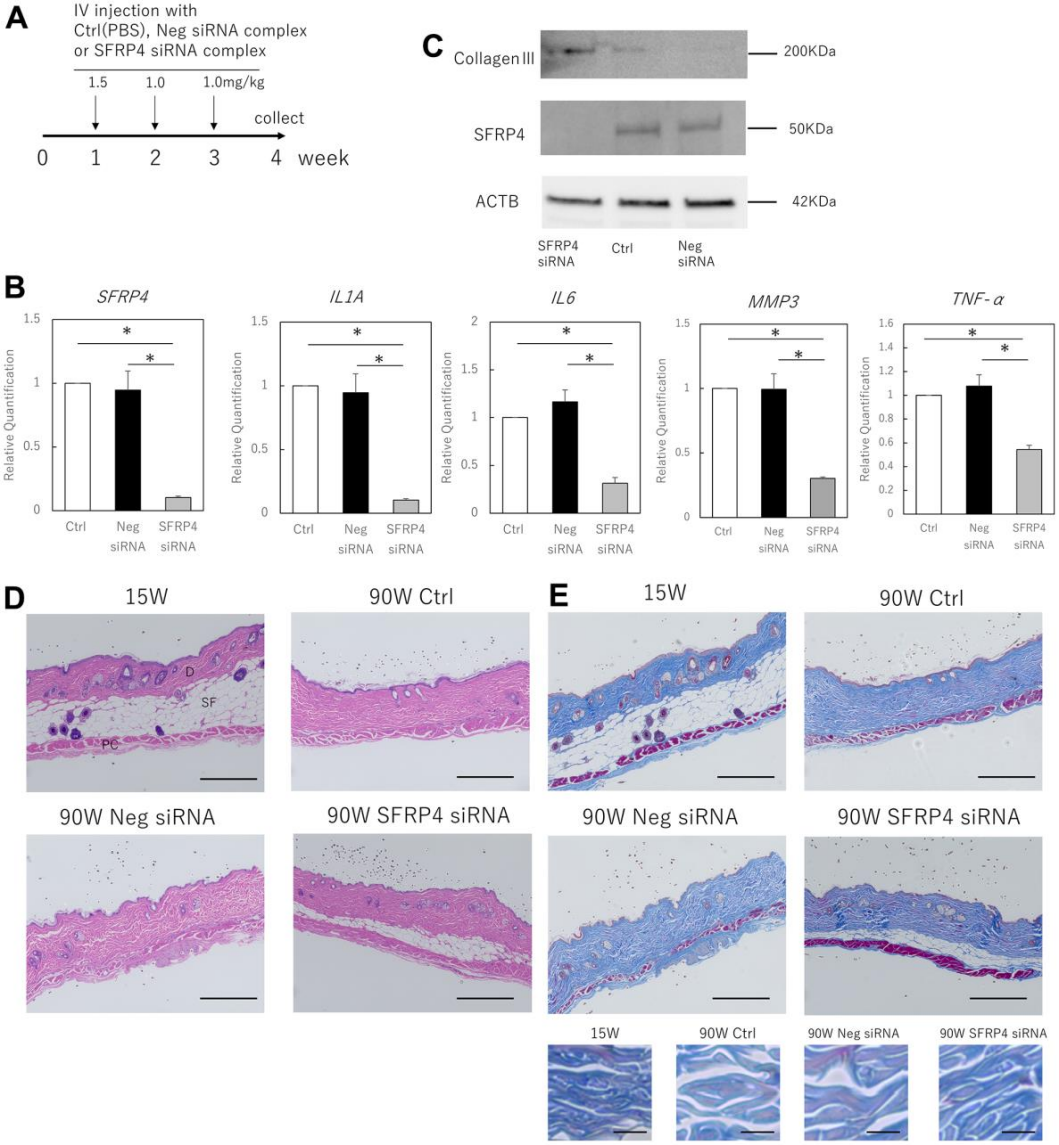
**Effects of SFRP4 knockdown on the skin of aging mice.** (**A**) Time course of the experiment. (**B**) RT-PCR analysis of SFRP4 and SASP gene expression in total skin extract. (**C**) Western blot analysis of collagen III and SFRP4 expression in whole skin extracts. SFRP4 siRNA treatment suppressed the expression of SASP factors and aging-related proteins, and increased collagen III. (**D**) H-E staining of skin from young (15 weeks old), control (PBS) aged, negative siRNA, and SFRP4 siRNA mice; thinning of subcutaneous adipose tissue and panniculus carnosus was observed in the control aged and negative siRNA groups. SFRP4 siRNA treatment improved these phenotypes. SF: Subcutaneous fat tissue; PC: Panniculus carnosus. Bar = 100 μm. (**E**) Skin in each intervention mouse; Masson’s trichrome staining; reduced thickness and density of collagen fibers and dermal connective tissue were observed in control aged mice and negative siRNA-treated mice, but the same improved with SFRP4 siRNA treatment. Collagen fibers are shown at higher magnification in the lower panel. Bar = 100 μm in the overall view, bar: 5 μm in magnified view. *; P < 0.05. Experiments were performed with n = 5 for each group. One-way ANOVA and Tukey’s post hoc test were used to compare differences between three groups.

We investigated the possibility that the *in vivo* inhibition of SFRP4 might inhibit SASP-induced skin aging in mice (Figure 4A). siRNA treatment with invivofectamine in mice resulted in a strong knockdown of SFRP4 in the skin (control vs. SFRP4 siRNA; P = 0.00036, negative siRNA vs. SFRP4 siRNA; P= 0.0063). Simultaneously, the gene expression of IL1A (control vs SFRP4 siRNA; P = 0.00026, negative siRNA vs SFRP4 siRNA; P = 0.0064), IL6 (control vs SFRP4 siRNA; P = 0.0018, negative siRNA vs SFRP4 siRNA; P = 0.0029), MMP3 (control vs SFRP4 siRNA; P = 0.0019, negative siRNA vs SFRP4 siRNA; P = 0.002), and TNF-α (control vs SFRP4 siRNA; P = 0.0021, negative siRNA vs SFRP4 siRNA; P = 0.012) was significantly reduced in the skin (Figure 4B). At the protein level, SFRP4 siRNA-treated skin also showed decreased SFRP4 expression, whereas the expression of collagen III, which is involved in dermal aging, was increased (Figure 4C). Histologically, the skin from mice in which SFRP4 was knocked down by siRNA showed increased subcutaneous adipose tissue and panniculus carnosus muscle fibers (Figure 4D). Masson’s trichrome staining also revealed that the collagen fibers in the dermis of negative siRNA and control mice were thin and loosely assembled, whereas those in SFRP4 siRNA mice were thick and dense, similar to those in young mice (Figure 4E). This indicates that the knockdown of SFRP4 in the skin of aging organisms improves the skin aging phenotype.

## DISCUSSION

This study shows that SFRP4, which is specifically expressed in aged p16^ink4a^-positive skin fibroblasts, contributes to SASP, and that treatment with SFRP4 causes worsening of this phenotype. To the best of our knowledge, the present study is the first to report that the suppression of SFRP4 expression *in vivo* ameliorates skin aging-related phenotypes, that is, adipose tissue atrophy and collagen fiber thinning, via SASP suppression.

With age, more senescent cells are observed in mouse and human tissues owing to an increase in senescent cells themselves and/or failure of clearance of such cells by apoptosis or immunity [23–27]. Long-term senescent fibroblasts enforce tissue decline and skin aging owing to their non-proliferative state with increased proteolytic activity and suppressed ability to deposit ECM components. In particular, skin fibroblasts with high p16^ink4a^ expression cause downregulated proliferation, likely due to the increased inhibitory effects on the CDK4/6 and retinoblastoma pathways, which leads to cell cycle arrest [28]. This SASP-promoted induction of inflammation by immortalized senescent cells clearly contributes to aging [13, 29]. In particular, an increase in p16^ink4a^-positive fibroblasts in the dermis has been reported to correlate with the typical morphological features of wrinkle formation and elastic fiber aging in human skin [30]. In this regard, clearance of p16^ink4a^ cells leads to a significant improvement in lifespan and organ function *in vivo* [21] and has been reported to improve the skin phenotype associated with aging [31]. In other words, the removal of senescent cells in aging skin or suppression of SASP may be an effective means to help develop therapies to antagonize skin aging.

In our study, we first observed SASP development and increased expression of SFRP4 in p16^ink4a^-positive fibroblasts. Furthermore, we found enhancement of SASP through SFRP4 ingestion and suppression of SASP through SFRP4 downregulation. However, there are few reports on the association between SFRP4 expression and aging. SFRP has garnered attention because it is highly expressed in fibroblasts and melanocytes near the basal layer of the epidermis and is involved in the pathogenesis of scleroderma, a type of immune fibrosis [25]. Its mechanism of binding to the Wnt ligand, which generally acts in negative regulation, not only has a complex effect on the formation of the Wnt gradient, but also extends the signaling range of the ligand [32, 33]. In particular, single-cell RNAseq revealed increased SFRP4 in different subgroups of fibroblasts isolated from systemic sclerosis interstitial lung disease (ILD) biosamples, which were hypothesized to be progenitor cells of myofibroblasts [34]. Even in normal skin, abnormal fibroblast activation and inadequate extracellular matrix deposition have been implicated in the aging skin phenotype [35], suggesting that SFRP4 expression in dermal fibroblasts may influence the skin phenotype associated with aging. However, the mechanisms by which SFRP4 is upregulated in dermal fibroblasts with aging are not fully understood, and should be investigated through comprehensive genetic and cascade analyses. For example, TGF-β, another mediator involved in fibrosis, has been shown to activate the canonical Wnt pathway and to potently stimulate fibroblast activation and tissue fibrosis [36], which may be in cross-talk with SFRP4.

Another mediator that affects dermal fibroblast damage in relation to aging is TNF-α. In this study, TNF-α, as one of the SASP characteristics of dermal fibroblasts, has been demonstrated to have catabolic activity in human skin through degradation of collagen I and upregulation of MMP [37]. Previous studies have shown that TNF-α-induced senescence is mediated by p38 MAPK and ROS, and the accumulation of senescent cells in aging skin and non-healing chronic wounds may play an important role in tissue remodeling, repair, and homeostasis regulation [38, 39]. The suppression of TNF-α expression and improvement of the aging skin phenotype by suppressing SFRP4 expression observed in this study may provide some clues to pursue the role of aging dermal fibroblasts in skin.

Since the skin is essentially atrophic in aging mice and this phenotype of skin atrophy has been repeatedly reported as comparable in humans [40, 41], we focused on the effects of SFRP4 knockdown on aging mouse skin. In particular, both collagen deposition and primary fiber thickness in aging mouse skin have been shown to be greatly reduced in the dermis [42], and suppression of SFRP4 expression achieved an improvement in this measure. Decreases in collagen III with aging have been also reported previously [42] suggesting that knockdown of SFRP4 has a protective effect on skin aging via collagen III increase and SASP suppression.

A limitation of this study is that other effects of SFRP4 knockdown on skin have not been fully examined; some parts of the SASP perform essential biological functions in wound healing and in response to short-term tissue damage [43, 44]. To avoid interference with the beneficial functions of the aging phenomenon, interventions targeting senescent cells in the context of aging and aging-related diseases may need to be temporally and locally particular to specific conditions. This can be accomplished through further studies of the specific mechanisms of suppression of senescent cell function using SFRP4 knockdown mice. These studies will ultimately lead to the development of new therapeutic strategies for age-related medical conditions. In addition, this study used only male mice to avoid any effects attributable to fluctuating hormones, but future experiments on female mice are needed to consider applications in humans.

Overall, this research could potentially aid in the development of anti-aging treatments for skin by preventing age-related changes through the regulation of SFRP4 expression in aging cells.

## MATERIALS AND METHODS

The study protocol was reviewed and approved by the Institutional Animal Care and Use Committee of our institution, the Keio University School of Medicine (approval number: 13072-(2)). All experiments were performed in accordance with the institutional guidelines on animal experimentation.

### Cell culture

Normal human dermal fibroblasts (C-12300) were obtained from PromoCell GmbH (Heidelberg, Germany). The cells were grown in low-glucose Dulbecco’s Modified Eagle Medium (DMEM; Wako Pure Chemical Industries, Osaka, Japan) supplemented with 10% fetal bovine serum (Thermo Fisher Scientific, Waltham, MA, USA) and 1% penicillin/streptomycin (Thermo Fisher Scientific). Fibroblasts with proliferative senescence or more than 2 weeks were defined as the absence of cell proliferation. Intracellular SA-β-gal activity was assessed using the Senescence β-Galactosidase Staining Kit from Cell Signaling Technology (Danvers, MA, USA).

### Assessment of BrdU incorporation in fibroblasts by flow cytometry

The cells were incubated with BrdU for 24 h at 37° C, then collected, and incubated with BrdU-FITC antibody (BrdUFlowEx FITC Kit; EXBIO Praha, a. s., Vestec, Czech Republic) for 30 min. A flow cytometer (BD Biosciences, Franklin Lakes, NJ, USA) was used for the analysis, and FlowJo (Ver. 10.2) was used to count the number of positive cells.

### Immunocytochemistry

The cells were placed on glass slides, fixed in acetone for 5 min at room temperature (15–25° C), and dried completely before staining. Cells were incubated overnight at 4° C with an anti-SFRP4 antibody (Thermo Fisher Scientific) and anti-p16^ink4a^ antibody (Abcam, Cambridge, UK) diluted at 1:100 in phosphate-buffered saline (PBS). After washing thrice with PBS, the slides were incubated with Alexa Fluor 488 conjugated goat anti-rabbit antibody and AlexaFluor555 conjugated donkey anti-goat antibody (Thermo Fisher Scientific) diluted at 1:2000 in PBS for 1 h at room temperature. After incubation, the slides were washed thrice with PBS and counterstained for nuclear visualization using ProLong Gold Anti-fade Mountant (Thermo Fisher Scientific) containing 4ʹ,6-diamidino-2-phenylindole.

### Treatment of human fibroblasts with recombinant human proteins

Aged human skin fibroblasts (PD55-60) were maintained in acclimated DMEM containing 15 μg/ml recombinant SFRP4 (rSFRP4; ab245787, Abcam). The rSFRP4-containing medium was supplemented every 2 days. Samples were analyzed for the induction of senescence after 10 days using the SA-β-galactosidase assay (SA-βGal) and typical senescence markers in a real-time PCR.

### RNA interference and transfection method

Lipofectamine 2000 (11668-019; Life Technologies, Invitrogen, Germany) was used to inject SFRP4 siRNA (121422, 121423, and 121424; Silencer™ siRNA, Thermo Fisher Scientific). RNA was collected from the cells after 72 h, and specific gene knockdown was assessed using real-time PCR.

### Western blotting

Total protein was extracted from the cells and tissues. Tissues were pre-shredded and treated with collagenase. Samples were extracted in lysate buffer: 50 mM Tris-HCl (pH 8.0), 150 mM NaCl, 0.5% Nonidet P40, 0.5% sodium deoxycholate, and phenylmethylsulfonyl fluoride (FUJIFILM Wako Pure Chemical Co., Osaka, Japan). Western blotting was performed as previously indicated [45]. Briefly, each sample (40 μg) was electrophoresed on 10% polyacrylamide gels Mini-PROTEAN® TGX™ Precast Gels (Bio-Rad Laboratories, Inc., CA, USA) and transferred to a Trans-Blot Turbo Transfer System (Bio-Rad).

After blocking with 3% nonfat milk for 2 h at room temperature, primary antibodies against SFRP4 (ab154167, Abcam. 1:200), p21 (ab220206, Abcam, 1:100), SIRT1 (ab32441, Abcam, 1:100), collagen III (PA5-27828, Thermo Fisher Scientific, 1:200), and GAPDH (1:2000 dilution; Santa Cruz Biotechnology, Santa Cruz, CA, USA) diluted in blocking solution were incubated overnight at 4° C. The next day, the sections were incubated with the following secondary antibodies: donkey anti-goat IgG H&L (HRP) (ab6885; Abcam), goat anti-rabbit IgG H&L (HRP) (ab205718; Abcam), and goat anti-mouse IgG H&L (HRP) (ab205719; Abcam) at 1:1000 dilution for 2 h at 37° C. After washing, the immunoreactive protein bands were visualized using an electrochemiluminescence detection kit (Pierce Biotechnology, Rockford, IL, USA). Images of the bands were obtained using a chemiluminescence imager (ImageQuant LAS4000 mini; GE Healthcare, Chicago, IL, USA). Image analysis was performed using ImageJ software (version 1.53p). Each experiment was repeated thrice.

### Performing the ELISA

Replicative senescent cell models were prepared as described above, and the medium was changed to serum-free medium containing antibiotics. After 24 h, the conditioned medium was collected and IL-6 and IL-8 expression was quantified using ELISA (Human IL-6 Quantikine ELISA Kit (D6050), Human IL-8 Quantikine ELISA Kit (D8000C), (R&D Systems, Inc., Minneapolis, MS, USA)), according to the manufacturer’s protocol.

### Animal experiments

Male C57BL/6 mice were purchased from the Sankyo Labo Service Corporation, Inc. (Tokyo, Japan). Young (15 weeks old) and old (90 weeks old) mice were both used in this study. The complexes were prepared by combining invivofectamine 2.0 (Life Technologies, Carlsbad, CA, USA) and SFRP4 siRNA (152089, 152090, and 152091; Silencer™ siRNA, Thermo Fisher Scientific), according to the manufacturer’s instructions. The SFRP4 siRNA complex, negative siRNA (Silencer™ Negative Control No. 1 siRNA, Thermo Fisher Scientific) complex, or control (PBS) were injected at 4 mg/kg into old mice by intravenous injection weekly (1.5 mg/kg in week 1 and 1 mg/kg in weeks 2 and 3). Experiments were performed with n = 5 for each group. One week later, the skin was collected in whole layers, and tissue specimens were fixed by immersion in 4% paraformaldehyde, embedded in paraffin, sectioned, and stained with hematoxylin-eosin and Masson’s trichrome staining. Tissues for RNA recovery were later immersed in RNA (Qiagen, Hilden, Germany) and stored at −20° C until use.

### RNA extraction and reverse transcription

Total RNA was extracted from cells or skin tissue using a monophasic solution of phenol and guanidine isothiocyanate (ISOGEN; Nippon Gene, Tokyo, Japan), according to the manufacturer’s instructions. Total RNA was mixed with random primers, reverse transcriptase, and dNTP mixture (Takara Bio Inc., Shiga, Japan). The mixture was incubated in a T100TM thermal cycler (Bio-Rad Laboratories, Inc., Hercules, CA, USA) at 25° C for 5 min, 55° C for 10 min, and 80° C for 10 min for heat inactivation of reverse transcriptase to generate cDNA.

### Real-time quantitative PCR (RT-qPCR)

RT-qPCR was performed using the Applied Biosystems 7500 Fast Real-Time PCR System (Thermo Fisher Scientific). A total of 40 cycles were performed and the fluorescence of each sample was measured at the end of each cycle. The PCR reaction was performed in two major steps: holding the reagents at 95° C for 3 s (denaturation) and at 60° C for 30 s (annealing and extension). In the subsequent melting curve analysis stage, the temperature was increased from 60° C to 95° C, and fluorescence was measured continuously. Gene expression was analyzed for *SFRP4* (Assay ID: Hs00180066_m1, Mm00840104_m1), *Il-6* (Hs00985639_m1, Mm00446190_m1), *Il-1a* (Hs00174092_m1, Mm00515166_m1), *Il-8* (Hs00174103_m1, Mm00441263_m1), *MMP3* (Hs00968305_m1, Mm00440295_m1) and *TNF-α* (Hs00174128_ml, Mm00443258_m1) (Thermo Fisher Scientific). PCR master mix (Cat. 4352042; Applied Biosystems, Foster City, CA, USA) was used, according to the manufacturer’s instructions; *GAPDH* (Hs02786624_g1) and *ACTB* (Mm02619580_g1) were used as control genes for normalization, according to the manufacturer’s instructions. Gene expression levels in the proliferating cell population were used as the baseline, and fold-change values were determined using the 2^−ΔΔCt^ method [46].

### Statistical analysis

Statistical analyses were performed using GraphPad Prism (version 5.0; San Diego, CA, USA) or SPSS 22.0 (Chicago, IL, USA). Mann–Whitney U test was used to analyze differences between two groups. One-way ANOVA and Tukey’s post hoc test were used to compare differences between three or more groups. Statistical significance was set at P < 0.05.

### Data availability statement

The data that support the findings of this study are available from the corresponding author, K.T., upon reasonable request.
